# Contrast-enhanced cine cardiac MR detects impairment of coronary microvascular perfusion in patients after acute myocardial infarct

**DOI:** 10.1186/1532-429X-11-S1-P269

**Published:** 2009-01-28

**Authors:** Ricardo Loureiro, Hiram G Bezerra, Joalbo M Andrade, Chun-Ho Yun, Ammar Sarwar, Michael Bolen, Thomas J Brady, Ricardo C Cury

**Affiliations:** 1 Massachusetts General Hospital – Harvard Medical School, Boston, MA USA; 2 Department of Radiology, Universidade de Brasília, Brasilia, Brazil

**Keywords:** Infarct Size, Microvascular Obstruction, Magnetic Resonance Imaging Measure, Myocardial Mass, Percutaneous Coronary Angioplasty

## Purpose

To prospectively compare contrast-enhanced (CE) cine steady-state free precession (SSFP) magnetic resonance imaging (MRI) to assess microvascular obstruction (MVO) with delayed-enhancement (DE) MRI, and to evaluate the association between MVO size and angiographic perfusion scores after primary percutaneous coronary angioplasty.

## Materials and methods

The study was HIPAA compliant and was approved by the institutional review board. All participants gave written informed consent. Twenty one patients (19 men; mean age, 59 ± 12 years) were examined with contrast-enhanced MRI after presentation and repeated at 6 months in 15 patients. TIMI Flow Grade (TFG), corrected TIMI Frame Count (cTFC), TIMI Myocardial Perfusion grade (TMPG), MVO, infarct size, and left ventricular ejection fraction (EF) were assessed.

## Results

MVO zones of decreased signal intensity were observed in 18 of 21 (86%) examinations on CE cineSSFP MR imaging and 17/21 (80%) examinations on DE MRI (P = NS). The size of CE cineSSFP hypointense zone was comparable to the MVO size measured by DE MRI (3.3 ± 3.1% versus 2.9 ± 3.0% of the myocardial mass; *P* = NS). MVO size correlated well with infarct size (r = 0.76 for CE cineSSFP and r = 0.83 for DE MRI) and EF at follow-up (r = -0.64 for CE cineSSFP and r = -0.58 for CE MRI). TFG 3 was present following percutaneous coronary intervention (PCI) in 20/21 (95%) patients. Abnormal post-PCI TMPG (0/1/2) was present in 11/21 (50%) of subjects and was associated with MVO size on CE cineSSFP (3.6 ± 2.2% with abnormal TMPG versus 0.8 ± 0.7% of the myocardial mass (LV) with TMPG 3; *P* < 0.05) and DE MRI (4.4 ± 2.6% with abnormal TMPG versus 1.3 ± 1.3% LV with TMPG 3; *P* < 0.05). There was no association between abnormal post-PCI TFC (≥ 40) and MVO size on CE cineSSFP (3.5 ± 2.5% with abnormal TFC versus 1.8 ± 2.3% LV with TFC < 40, P = 0.26) and DE MRI (2.9 ± 2.0% with abnormal TFC versus 1.5 ± 2.3% LV with TFC < 40, P = 0.29). Abnormal post-PCI TMPG was also associated with greater relative infarct size (18.8% vs. 9.6%, P = 0.04). CEcineSSFP MR had a comparable contrast-to-noise ratio (CNR) to DE MRI for depicting MVO zones (P = NS).

## Conclusion

CE cineSSFP hypointense zone depicts the microvascular obstruction region in close agreement with the DE MRI approach, and correlates with infarct size and global LV function at follow-up. TMPG seems to translate better the microvascular integrity compared to cardiac MRI measures of MVO.

